# Hydrogel-Based Stimuli-Responsive Micromotors for Biomedicine

**DOI:** 10.34133/2022/9852853

**Published:** 2022-10-07

**Authors:** Huaijuan Zhou, Guozhao Dong, Ge Gao, Ran Du, Xiaoying Tang, Yining Ma, Jinhua Li

**Affiliations:** ^1^Advanced Research Institute of Multidisciplinary Sciences, Beijing Institute of Technology, Beijing 100081, China; ^2^School of Medical Technology, Beijing Institute of Technology, Beijing 100081, China; ^3^School of Materials Science & Engineering, Key Laboratory of High Energy Density Materials of the Ministry of Education, Beijing Institute of Technology, Beijing 100081, China; ^4^School of Life Science, Beijing Institute of Technology, Beijing 100081, China; ^5^Department of Forensic Science, Jiangsu Police Institute, Nanjing 210031, China

## Abstract

The rapid development of medical micromotors draws a beautiful blueprint for the noninvasive or minimally invasive diagnosis and therapy. By combining stimuli-sensitive hydrogel materials, micromotors are bestowed with new characteristics such as stimuli-responsive shape transformation/morphing, excellent biocompatibility and biodegradability, and drug loading ability. Actuated by chemical fuels or external fields (e.g., magnetic field, ultrasound, light, and electric field), hydrogel-based stimuli-responsive (HBSR) micromotors can be utilized to load therapeutic agents into the hydrogel networks or directly grip the target cargos (e.g., drug-loaded particles, cells, and thrombus), transport them to sites of interest (e.g., tumor area and diseased tissues), and unload the cargos or execute a specific task (e.g., cell capture, targeted sampling, and removal of blood clots) in response to a stimulus (e.g., change of temperature, pH, ion strength, and chemicals) in the physiological environment. The high flexibility, adaptive capacity, and shape morphing property enable the HBSR micromotors to complete specific medical tasks in complex physiological scenarios, especially in confined, hard-to-reach tissues, and vessels of the body. Herein, this review summarizes the current progress in hydrogel-based medical micromotors with stimuli responsiveness. The thermo-responsive, photothermal-responsive, magnetocaloric-responsive, pH-responsive, ionic-strength-responsive, and chemoresponsive micromotors are discussed in detail. Finally, current challenges and future perspectives for the development of HBSR micromotors in the biomedical field are discussed.

## 1. Introduction

Micromotors refer to mobile miniaturized machines with sizes ranging from several micrometers to hundreds of micrometers [1–7]. The great potential of applying micromotors in the biomedical field, especially its great promise in targeted drug delivery [8, 9], precision medicine [10], noninvasive diagnosis, and minimally invasive surgery [11–13], has attracted many researchers to join in this emerging multidiscipline. In literature, researchers have given micromotors many interesting names such as microrobots [14], microgrippers [15], microactuators [16], microswimmers [17], microwalkers [18], microrockets [19], and microbowls [20] according to their functionality, motion mode, and geometric shape [21]. Compared to stationary and passive nanomaterials, autonomous or controlled locomotion ability is the most distinctive feature of micromotors. Both chemicals and external fields (magnetic field, light, ultrasound field, electric field, etc.) can be the propulsion sources to drive the movement as long as the micromotors possess relevant components in response to these sources [22–25]. Accordingly, micromotors can be classified as bubble-propelled micromotors [26], magnetically driven micromotors [27–30], light-driven micromotors [31, 32], ultrasound-driven micromotors [33], and so forth [34].

The hydrogel-based stimuli-responsive (HBSR) micromotors are normally composed of (i) stimuli-responsive (e.g., thermo-sensitive, photothermal-sensitive, pH-sensitive, ion-strength-sensitive, and chemosensitive) hydrogels, (ii) actuation-responsive materials, and (iii) other functional components (e.g., drugs). The stimuli-responsive hydrogels enable the HBSR micromotors to perform a displacement-free action (e.g., unfolding and self-folding behaviors, which are similar to the grasping and releasing functions of hands) upon the change in environmental parameters, such as temperature, pH, and ion strength [35]. The actuation-responsive materials (e.g., magnetic nanoparticles, noble metal catalysts, and enzyme catalysts) are the materials in response to chemicals and external fields, which enable the HBSR micromotors to perform a propulsion action (e.g., locomotion in a physiological environment to transport a target cargo under the navigation of magnetic field). Indeed, both displacement-free actions and propulsion actions are significantly important for practical biomedical applications since a series of actions are involved to complete a specific medical task in a complicated physiological environment. Taking targeted cancer therapy as an example, the HBSR micromotors are supposed to have the fundamental abilities to autonomously load anticancer drugs, transport the therapeutic reagents to tumor sites, and release the drugs by the combined utilization of stimuli and actuation sources (e.g., magnetic field, bubble propulsion, and ultrasound).

**Scheme 1:**
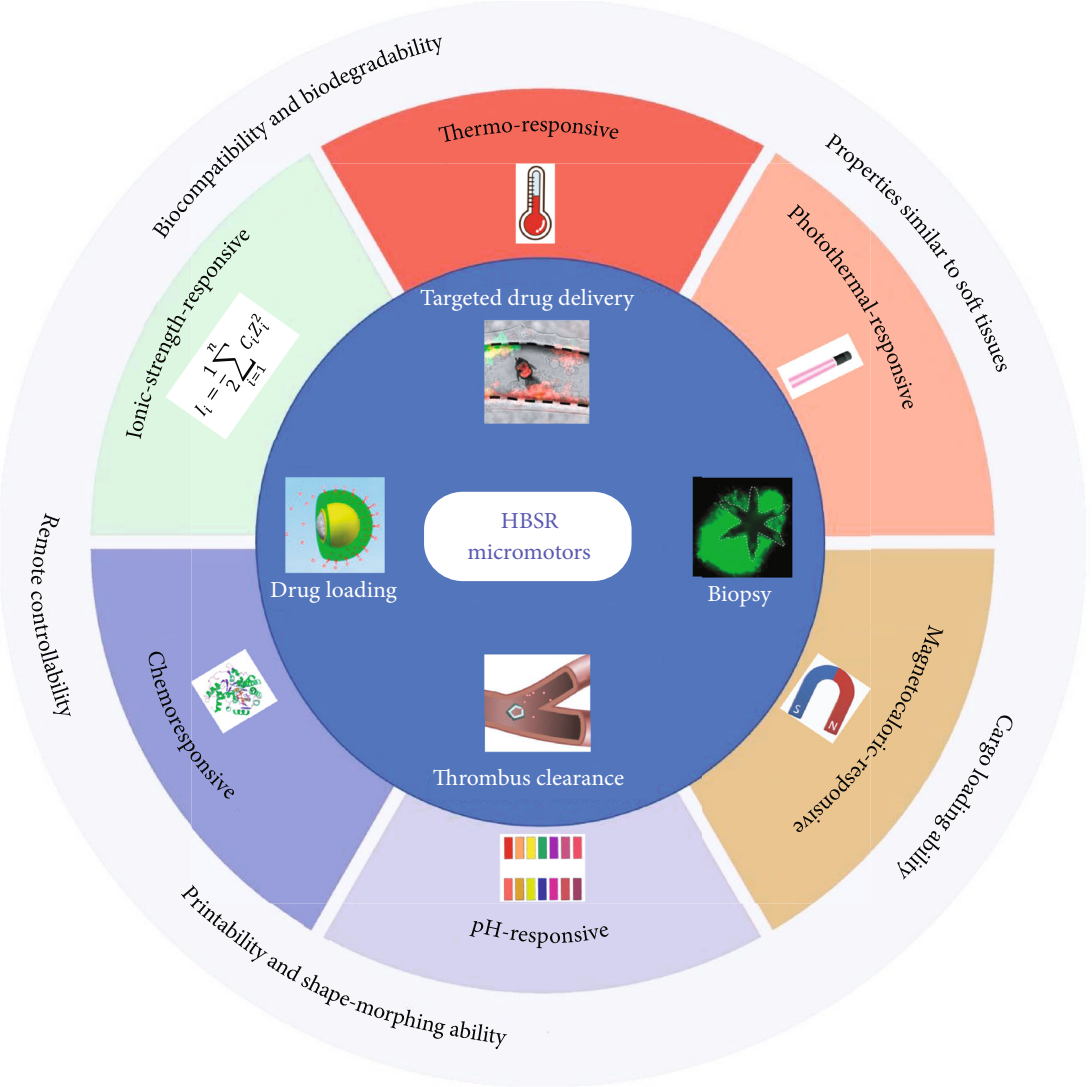
Classification, advantages, and applications of hydrogel-based stimuli-responsive (HBSR) micromotors.

Scheme 1 presents the classification of HBSB micromotors according to the type of stimulus and summarizes the advantages and biomedical applications of HBSB micromotors. More specifically, the great potential of applying hydrogel-based micromotors in the biomedical field lies in the following advantages:

1. Biocompatibility and biodegradability. Most hydrogels, especially natural polymer-based hydrogels (e.g., gelatin, collagen, hyaluronic acid, and heparin) are biocompatible and biodegradable, which ensures that the HBSR micromotors do not cause toxicity or other side effects to the host body and can spontaneously degrade or be retrieved from the physiological environment after they fulfill their tasks, such as transport, therapy, and diagnosis
2. Properties similar to soft tissues. Hydrogels, which can store a large number of water molecules inside their three-dimensional (3D) network, have properties similar (e.g., mechanical property, chemical property, biological property, and ion transport capacity) to soft tissues. Hydrogel-based micromotors can inherit the versatile properties of their parent materials at multiple scales
3. Cargo loading ability. Therapeutic drugs, nanoparticles, and even cells with suitable sizes can diffuse in or out of the hydrogel matrices due to the 3D network structure of hydrogels. For instance, the stimuli-responsive micromotors can load therapeutic reagents (e.g., antitumor drugs) to the network of hydrogel layer, then transport them to a targeted diseased region under the actuation of chemical fuels or external fields, and finally expel the reagents out of the network upon the stimuli to execute their therapeutic tasks
4. Printability and shape-morphing ability. Hydrogel-based micromotors with various geometric structures including complex architectures can be achieved by using 3D printing [21, 36–40], 4D printing [41], direct laser writing [42], and other techniques due to the excellent processability of most hydrogel materials. Furthermore, shape-morphing is a distinctive feature of soft micromotors [43], which enables the micromotors to adapt to variational physiological surroundings and move to the hard-to-reach sites or spaces
5. Remote controllability. Different from the random locomotion of self-propelled micromotors, the movement behaviors (including motion velocity, direction, and path) of field-driven micromotors can be remotely governed by the magnetic field, light, ultrasound, electric field, and other external fields. Combined with imaging systems (such as magnetic resonance imaging, ultrasound imaging, X-ray computed tomography, and other imaging techniques in clinical practice), the whole movement and task implementation process of micromotors can be monitored and remotely controlled in a real-time manner

## 2. HBSR Micromotors and Biomedical Applications

An intelligent medical micromotor is supposed to have the ability to sense environmental variation and promptly respond to the disturbance. For example, initially, a micromotor with cargos (e.g., therapeutic drugs) in its hands aimlessly wanders in the biological environment. When it meets with a tumor tissue or abnormal object by sensing the changes in local pH, temperature, chemical composition, or other clues, it reacts immediately by releasing the cargo in the anomalous area. If the diseased area has been clinically positioned with the assistance of medical imaging system, the micromotors can be actuated and navigated directly along a designed path and finally reached the targeted sites for disease treatment. According to the category of triggers, this review summarizes the current research on hydrogel-based thermo-responsive, photothermal-responsive, magnetocaloric-responsive, pH-responsive, ionic-strength-responsive, and chemoresponsive micromotors. Representative micromotors and their (potential) biomedical applications are introduced to allow readers to gain insight into the latest developments in this emerging discipline.

### 2.1. Hydrogel-Based Thermo-Responsive Micromotors

The incorporation of thermo-responsive hydrogels empowers micromotors with the thermo-sensitive property. Thermo-responsive hydrogels possess hydrophobic groups and hydrophilic groups in their chemical structure. Poly(N-isopropylacrylamide) (often abbreviated as PNIPAM, also known as PNIPA, PNIPAAm, NIPA, PNIPAA, or PNIPAm) hydrogel is the most commonly used thermo-responsive material due to its advantages of excellent biocompatibility, good degradability, and suitable phase transition temperature (close to human body temperature) [44]. It presents a sharp phase transition and a large swelling ratio at around 33°C. Such a transition temperature is called lower critical solution temperature (LCST). For PNIPAM, its LCST can be adjusted slightly near the human body temperature by modifying the ratio of the hydrophilic components to hydrophobic components (e.g., by copolymerization with hydrophilic monomers such as acrylic acid). When the temperature is lower than LCST, the hydrogel can absorb water molecules and swell. When the temperature is higher than LCST, however, the hydrogel shrinks and expels the water due to the change of molecular configuration. Taking advantage of this reversible swelling-shrinking shape transformation, actions of gripping and releasing can be achieved when thermo-responsive hydrogel is the main component of micromotors. Furthermore, such a shape change can be used to fabricate tubular micromotors via the spontaneous rolling-up process, and the velocity of the micromotors can be regulated by the temperature [45] .

**Figure 1:**
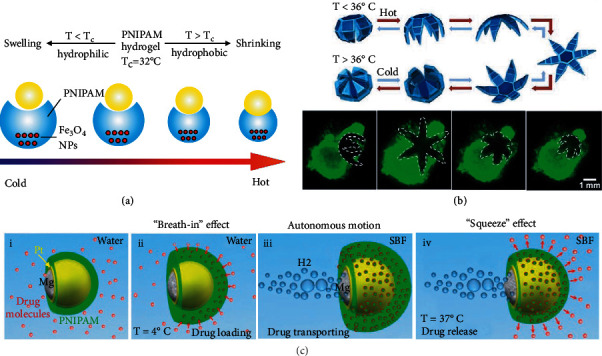
Representative hydrogel-based thermo-responsive micromotors. (a) A microgripper shrank and grasped the cargo when the temperature was above LCST. (b) Schematic image of a thermo-responsive microgripper exhibiting reversible self-folding and unfolding architectures (top); application of a thermo-responsive microgripper in capturing and excising cells from a live cell fibroblast clump (below). Reproduced with permission from ref [50]. Copyright 2015 American Chemical Society. (c) A schematic image of an Mg/Pt-PNIPAM Janus micromotor demonstrating the process of drug loading, transporting, and releasing behaviors. Reproduced with permission from ref [54]. Copyright 2014 American Chemical Society.

Xu's group [46] has developed a Janus micromotor via a one-step gas-liquid emulsion method. The presence of PNIPAM body enables the micromotors to grasp the cargo when the temperature is lower than LCST, demonstrated in Figure 1(a). However, the obvious drawback of this micromotor with such a simple geometrical shape lies in its poor operability dimension and limited opening width and strength for gripping action. Hence, more advanced thermo-responsive micromotors, especially microgrippers [47], have been developed when researchers attempted to realize more degrees of freedom in object operation at the microscopic size. For example, after modifying the surface of stomatocyte-like micromotors with the PNIPAM polymer brushes, the opening of bioinspired micromotors can be enlarged or narrowed by the stimulation of temperature. As a result, the locomotion of micromotors can be governed by temperature because thermo-responsive polymer can function as a valve or brake to regulate the access of hydrogen peroxide fuel [48]. Kuo et al. [49] fabricated a magnetic microgripper made from PNIPAM hydrogel with dispersed superparamagnetic Fe_3_O_4_ nanoparticles (NPs) and multiwall carbon nanotubes (MWCNTs) inside. The presence of MWCNTs could shorten the thermal response time of hydrogel due to the enhanced mass transport of water molecules. The addition of Fe_3_O_4_ NPs not only allows for the alignment by magnetization effect (i.e., form of chains along the direction of the applied magnetic field), but also enables a propulsive movement (i.e., translational motion and rotational motion) under the wireless manipulation of uniform magnetic fields. To achieve a gripping action, the micromotors were constructed by a bilayer hydrogel composite with different degrees of cross-linking. The gripping motion (similar to the behavior of tweezers) reached a full stroke at around 38°C by applying alternating current magnetic fields, which demonstrated the potential in drug delivery or assistance of minimally invasive surgery in the blood vessel. However, the HBSR microgripper with simple architecture often exhibits limited gripping ability due to the low modulus of thermo-responsive hydrogels, which restrains their ability to process the assigned tasks (such as pick-and-place a targeted cargo, surgical excision, etc.).

To further increase the feasibility of operation, hydrogel-based microgrippers with six arms have been developed [50, 51]. A six-arm microgripper was constructed by using a soft thermo-responsive PNIPAM-AAc hydrogel actuation layer with a low modulus (162 KPa) and nonswellable PPF polymer segments with a high modulus (16 MPa) (Figure 1(b)) [50]. After the incorporation of iron oxide, the magnetic HBSR microgripper is able to move to the targeted sites under the actuation of external magnetic fields and then excises required cells from a tissue clump due to the self-folding characteristics of temperature-responsive PNIPAM-AAc hydrogel upon the temperature stimuli and residual stress difference between the hydrogel and PPF polymer. Furthermore, the introduction of stiff PPF polymer endows the micromotor with sufficient strength to execute the task of cell excision for biopsy [50]. Go et al. [52] have developed thermo-electromagnetically actuated microgrippers with eight arms by using polyethyleneglycol diacrylate (PEGDA) with dispersed iron oxide NPs as the electromagnetically actuated layer and N-isopropylacrylamide (NIPAAM) as the thermo-responsive layer. The pulling action at the unfolded state of the HBSR micromotor and rolling action at its folded state endow the thermo-electromagnetically driven microgrippers with the behaviors of trapping, delivering, and releasing. In vitro experiments confirmed the feasibility of using such eight-arm micromotors for tumor therapy [52].

In addition to external fields, the driving force from the chemical fuels can also cause a propulsive movement of HBSR micromotors. Pt is a widely used material for self-propelled micromotors since it can generate oxygen bubbles via the decomposition of hydrogen peroxide (i.e., $H_{2}O_{2}\overset{Pt}{⟶}H_{2}O+O_{2}$). Considering the cost of Pt noble metal, researchers have tried to use other materials (e.g., Mg [53, 54] and Mn_2_O_3_ [55]) as alternatives for bubble-propelled micromotors. Guan's group has developed Mg/Pt-PNIPAM Janus micromotors, demonstrating autonomous motion in simulated body fluids (SBF) or blood plasma without any other additives [54]. As shown in Figure 1(c), when the environmental temperature is at 4°C, PNIPAM hydrogel shells of the HBSR micromotors have the capacity to encapsulate drug molecules via the temperature-induced “breath-in” effect. Transport of drugs can be achieved via the autonomous movement of micromotors, driven by the hydrogen bubbles from the reaction between Mg and H_2_O (i.e., Mg + H_2_O → Mg(OH)_2_ + H_2_). When the temperature reaches body temperature (i.e., 37°C), the drugs can be smoothly released from the HBSR micromotors due to the “squeeze” effect from the volume change of PNIPAM hydrogel shells.

### 2.2. Hydrogel-Based Photothermal-Responsive Micromotors

In addition to a direct heat source supplied by a heater, the temperature change induced by light, magnetic field, or other sources can also actuate the phase transition of thermo-responsive hydrogels. Near-infrared (NIR) light is attractive for biomedical applications since body tissue has the highest transmissivity in the NIR region (so-called “biological window”) and NIR almost has no side effects on human organs and tissues.

**Figure 2:**
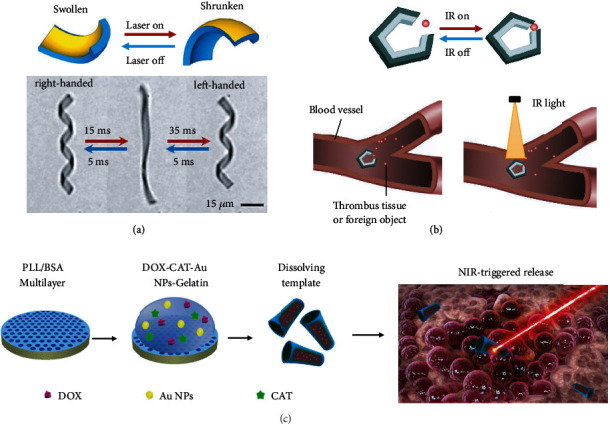
Representative hydrogel-based photothermal-responsive micromotors. (a) Fast reversible swelling/deswelling deformation of a microribbon (with gold nanorods inside) induced by the 808 nm laser irradiation. Reproduced with permission from ref [57]. Copyright 2017 American Chemical Society. (b) Schematic image of the actuation of a microgripper to remove thrombus tissue or foreign object in the blood vessel by the IR light irradiation. (c) Fabrication process and light-induced drug release of gelatin-based microrockets. Reproduced with permission from ref [59]. Copyright 2015, American Chemical Society.

In order to generate a photothermal effect, noble metal (e.g., Au and Ag) NPs are often incorporated into the composition of thermo-responsive micromotors because the localized surface plasmon resonance (LSPR) phenomenon of noble metal NPs can generate light-induced plasmonic heating [56–58]. Light-induced transformation deviates from the equilibrium transition path, demonstrating a fast response time (within microseconds) [57]. As shown in Figure 2(a), a bilayer microribbon with embedded gold nanorods in its PNIPAM layer changed its shape from a right-handed helix to an uncoiling state within 15 ms and then transformed from the uncoiling state to a left-handed helix within 35 ms by applying 808-nm laser for the plasmonic heating. It is worth noting that the temperature (i.e., water temperature) of the microribbon in this experiment was kept at 20°C, which was much lower than the LCST of its PNIPAM thermo-responsive layer. When the laser was off, fast recovery from the left-handed helix to the right-handed helix occurred upon cooling [57]. Jia et al. [58] reported that the temperature of HBSR microgrippers with dispersed gold nanorods in a PNIPAM layer can be raised above LCST within 5 s by the NIR laser irradiation since gold nanorods with a longitudinal surface plasmon resonance peak at 785 nm can transduce light into localized heat. Combined with the actuation control by external fields, such a photothermal-responsive microgripper can be used to complete cooperative missions in a confined space, such as removing thrombus tissue or foreign object in the blood vessel (Figure 2(b)). Wu et al. [59] have fabricated a self-propelled microrocket composed of bovine serum albumin/poly-L-lysine (BSA/PLL) multilayer, catalase, gelatin hydrogel, gold nanoparticles, and doxorubicin drugs via a template-assisted layer-by-layer assembly method. Upon NIR irradiation, the heat generated from the plasmonic gold nanoparticles caused the phase transition of the gelatin hydrogel, leading to the fast release of drugs for killing surrounding cancer cells (Figure 2(c)). The HBSR microrockets are biodegradable since the main composition are proteins and polypeptides, which can be degraded by enzymes in the human body [59]. Except for noble metal NPs, single-walled carbon nanotubes (SWCNT) have also been used for photothermal-responsive micromotors since SWCNT can efficiently generate heat by infrared light irradiation [60]. Furthermore, the accumulation of heat from prolonged NIR irradiation can also result in the deswelling motion of thermo-responsive hydrogels. Based on this mechanism, a helical micromotor was developed for precise targeting and active drug release [61]. The movement of micromotors with magnetic NPs and loaded therapeutic reagents was wirelessly controlled by an electromagnetic actuation system. After arriving at the targeted lesion, the HBSR micromotor containing alginate/NIPAM hydrogels shrank and released the drugs to kill the tumor (i.e., human hepatocellular carcinoma cells) when exposed to the NIR irradiation with the intensity of 1.5 W/m^2^ for 5 min [61].

### 2.3. Hydrogel-Based Magnetocaloric-Responsive Micromotors

The change of temperature induced by magnetic fields can also cause the thermo-responsive behaviors of HBSR micromotors. However, there are very limited reports on the shape change of thermo-responsive magnetic micromotors induced by the magnetothermal effect. Tabatabaei et al. [62] fabricated an HBSR micromotor composed of thermo-responsive NIPAAM hydrogels, magnetic NPs, and drugs. Controlled drug release was achieved through the shrinking behavior of the NIPAAM hydrogel, caused by the local temperature increment of magnetic nanoparticles after employing alternating magnetic fields.

### 2.4. Hydrogel-Based pH-Responsive Micromotors

The variations in pH are ubiquitous in certain body sites (such as vagina, gastrointestinal tract, and blood vessels), which provides a natural initiator to trigger the swelling/deswelling action of hydrogel-based pH-responsive micromotors. Scientists have successfully enhanced the sperm motion capacity and triggered sperm capacitation, which is a crucial maturation process before fertilization, by releasing heparin from HBSR micromotors containing pH-responsive gelatin hydrogels at pH 8 [63]. Based on the correlation between the pH value and the velocity of the bubble-propelled micromotor, pH-responsive HBSR micromotor containing gelatin hydrogels can be applied for motion-based pH sensing [64]. Moreover, in situ cell manipulation was reported by using pH-triggered soft microactuators [65]. The targeted drug delivery for cancer therapy was achieved by using a soft HBSR micromotor consisting of a bilayer hydrogel structure of 2-hydroxyethyl methacrylate (pH-responsive) and poly (ethylene glycol) acrylate with magnetic NPs [66].

**Figure 3:**
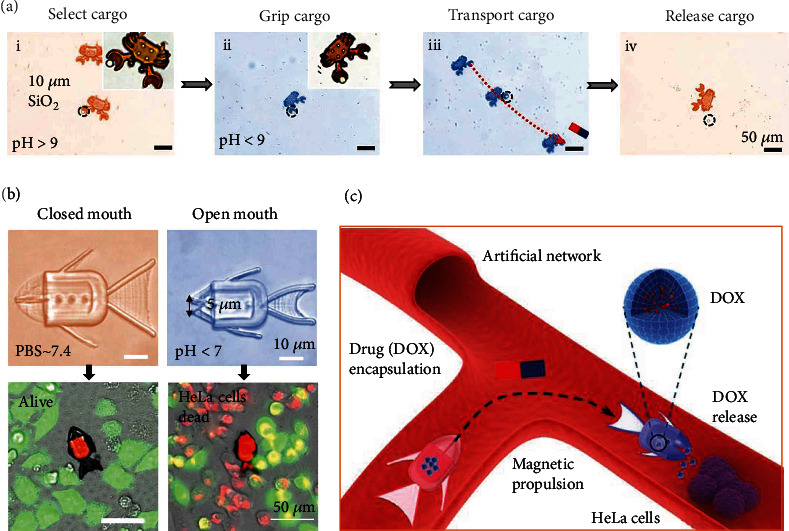
Representative hydrogel-based pH-responsive micromotors with the shape-morphing ability for cancer therapy. (a) Time-lapse optical images of a crab-like micromotor performing the tasks of targeted cargo selection, gripping induced by the change of environmental pH, magnetically-navigated transport, and controlled cargo release. (b) HeLa cells around a fish-like micromotor with tumoricidal drugs inside are alive after incubation for 6 hours since the micromotor closes its month in PBS, while many HeLa cells around the fish-like micromotor are dead since the micromotor opens its mouth at pH < 7, leading to the release of drugs from its body. (c) Schematic illustration of a shape-morphing micro-fish for targeted drug transport and controlled release under the navigation of magnetic field and the stimuli of pH change, respectively. Reproduced with permission from ref [68]. Copyright 2021 American Chemical Society.

Inspired by the biological systems that can dynamically change their shape and functions to adapt to the environmental variations, researchers have attempted to design and fabricate pH-responsive flexible micromotors with the shape-morphing ability [67]. Programmable shape morphing is realized by regulating the expansion rate of constructed components at a localized part of HBSR micromotors. A crab-like HBSR micromotor containing pH-sensitive poly(acrylic acid) (PAAc) hydrogel was fabricated by using femtosecond laser direct writing technology. Combined with magnetic actuation, the HBSR microcrab can grip, transport, and release cargo by making use of the opening and closing of its claw upon sensing the pH stimuli (Figure 3(a)) [68]. The carboxyl groups in PAAc hydrogel become deprotonated and negatively charged in a high pH environment. The electrostatic repulsion forces between molecular chains lead to significant expansion of PAAc hydrogel network. As a result, the HBSR micromotor opens its claw at pH>9. Inversely, at a pH<9, the HBSR micromotor closes its claw due to the shrinkage behavior of hydrogel from its network collapse. The closing/opening actions for cargo gripping and releasing can be repeated by the reversible protonation/deprotonation process. However, the responsiveness of such a crab-like HBSR micromotor at pH 9 limits its application under physiological conditions (pH ∼7.4). Hence, researchers fabricated a fish-like HBSR micromotor (Figure 3(b)), which can adjust its mouth shape between pH ∼7.4 and pH <7. This pH range is chosen because some tumor cells (e.g., HeLa cells) maintain good biological viability in a solution environment with pH <7. At pH < 7, the microfish with opened mouth can inhale the drugs (DOX) into its hollow body by the capillary force (i.e., drug loading process). After adding PBS solution at pH ~ 7.4, the HBSR microfish closes its mouth to encapsulate the drugs, aiming to avoid the release loss of drugs during the transportation process (i.e., pH-induced drug encapsulation process). When the HBSR microfish is delivered to the position of HeLa cell clusters with a local pH < 7 by the magnetic actuation and navigation, the HBSR microfish opens its mouth again to release the drugs for cancer therapy (i.e., pH-induced drug release process) as demonstrated in Figure 3(c). This research work demonstrates the high programmability of hydrogel-based microarchitectures by 4D printing techniques and reversible shape-morphing capacity of HBSR micromotors in response to a localized environment variation (i.e., pH values), which hold great promise for the targeted cancer therapy in a remote, wireless, efficient, and controllable manner.

### 2.5. Hydrogel-Based Ionic-Strength-Responsive Micromotors

**Figure 4:**
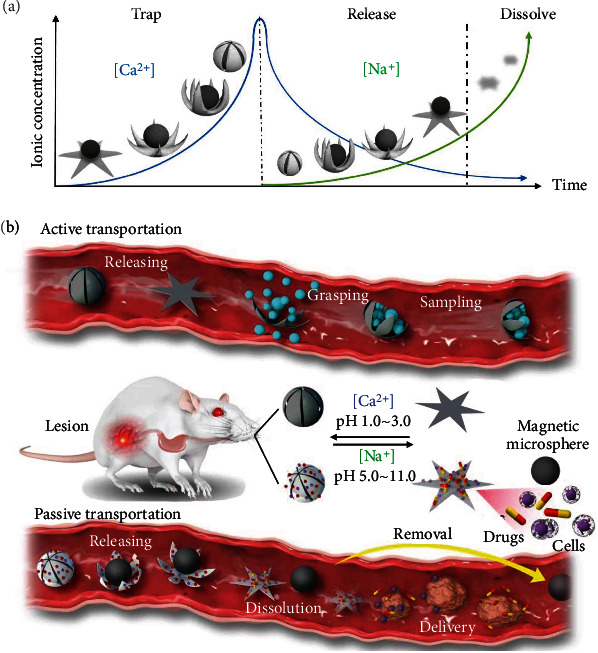
Representative hydrogel-based ionic-strength-responsive micromotors. (a) Illustration of the shape transformation process of a hydrogel-based microgripper under ionic stimulation. The microgripper self-traps a magnetic microsphere as the increase of Ca^2+^ concentration in the hydrogel via the application of a CaCl_2_ solution. The magnetic microsphere is released as Ca^2+^ is replaced by Na + via the addition of a sodium citrate solution. Finally, the microgripper was fully dissolved. (b) Schematic illustration of a hydrogel-based micromotor in the gastrointestinal tract with two transportation modes. Reproduced with permission from ref [68]. Copyright 2021 The Authors.

Ionic-strength-responsive micromotors can be constructed by using ionic-strength-responsive hydrogels, which must contain ionizable groups in their structure. The reports on ionic-strength-responsive micromotors are very limited. Taking advantage of the pH responsiveness and ionic sensitivity of alginate hydrogel, Zheng et al. [68] have developed a dual-responsive micromotor. The shrinkage and swelling actions of alginate-based microgrippers can be controlled either by pH value or the ion-exchange process between Na^+^ ions and multivalent cation (especially Ca^2+^). It is worth noting that these ions are widely distributed in the body fluid and they have almost no side effects as long as their concentrations do not exceed their respective safety threshold. The stiffness of the alginate hydrogel is in positive correlation with the concentration of Ca^2+^. High concentration of Ca^2+^ results in a high modulus of hydrogel because of more alginate-Ca^2+^ crosslinks and vice versa. Thanks to the inhomogeneous gel network density in the dual-responsive micromotor, an inhomogeneous shape deformation can be produced due to in-plane stress from the local differences in cross-linking density upon the change of pH or ionic strength. As shown in Figure 4, for an active transportation mode, the micromotor with embedded magnetic NPs can actively open and close its hands to grasp objects (e.g., cells, tissues) for sampling under the stimuli of ionic or pH. For a passive transportation mode, however, the locomotion of an HBSR micromotor with loaded payloads (e.g., cells, drugs) can be driven by external magnetic fields after the micromotor holds a magnetic microsphere in its hand. The payloads can be released from the HBSR micromotor for treating diseases or tumors when its surrounding environment has a pH of 5~11 (e.g., pH is around 7 in the intestinal tract) or it is exposed to a solution with Na^+^. The ex vivo experiments in a gastrointestinal tract by using an intestine extracted from a Sprague Dawley rat have confirmed the feasibility and versatility of such a dual-responsive HBSR micromotor for grasping and releasing an object on a microscopic scale under the stimuli of enteric pH or ionic strength [68].

### 2.6. Hydrogel-Based Chemoresponsive Micromotors

Chemoresponsive micromotors can form shape deformations by using specific chemicals, enzymes, or biological signals [69–72]. A fish-like micromotor, which is composed of PNIPAM hydrogel modified with phenylboronic acid and sodium dodecyl sulfate (a surfactant), was fabricated by Dong and coworkers [69]. The complexation between phenylboronic acid and glucose can cause the release of the surfactant attributed to the volume expansion of the HBSR micromotor, leading to the propulsion of the whole structure based on the so-called Marangoni effect. According to the characteristics of glucose-dependent motion and magnetically controlled motion, such a fish-like HBSR micromotor can be used to determine the glucose concentration in human serum or urine, which can find its practical application in detecting and monitoring diabetes [69].

**Figure 5:**
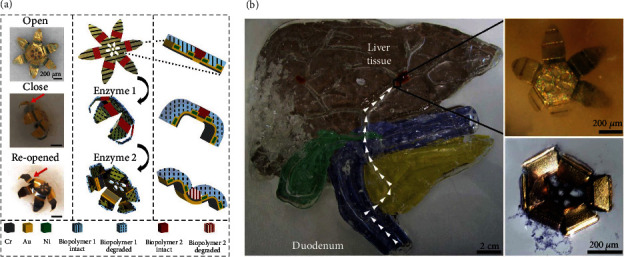
Representative hydrogel-based chemoresponsive micromotors. (a) Schematic illustration of a multilayered microgripper with integrated biopolymer layers which can close and reopen upon exposure to two different enzymes. (b) Magnetic navigation of a hydrogel-based enzyme-responsive micromotor to excise liver tissue for biopsy after insertion of cellulase. Reproduced with permission from ref [72]. Copyright 2010, American Chemical Society.

Enzymes have also been utilized to induce the shape transformation of HBSR micromotors. For example, a multilayered microgripper with alternating rigid segments (i.e., Cr, Au, and Ni) and flexible hinges (i.e., biopolymers) was fabricated by Bassik et al. [72]. The rigid segments provide the micromotor with enough mechanical strength during the cycle of closing and reopening, while two initially flat flexible hinges (made from gelatin and carboxymethylcellulose, respectively) can be bent with either concave or convex curvatures under the stimuli of appropriate enzymes. It is worth noting that gelatin can be naturally degraded by enzymes present in the disease area (e.g., proteases in cancer), which offers practical feasibility for autonomous actuation on exposure to a lesion area. However, carboxymethylcellulose can only be degraded by nonmammalian enzymes, indicating its noninteraction with animal tissue. The closing and reopening of the abovementioned multilayered HBSR microgripper is demonstrated in Figure 5(a). Biopolymer 1 is only sensitive to enzyme 1, while biopolymer 2 is only responsive to enzyme 2. When biopolymer 1 is selectively degraded by enzyme 1, the microgripper will close its hands, caused by the residual stress difference between the two biopolymers since the modulus of biopolymer 1 is decreased upon degradation while that of biopolymer 2 is intact. Subsequently, the micromotor can reopen its hands when the modulus of the biopolymer 2 is decreased upon exposure to enzyme 2, leading to the bending of the second hinge in the opposite position. Simulation experiments for biopsy were carried out in a model organ with a size close to that of an adult human, as shown in Figure 5(b). The enzyme-responsive microgripper in the duodenum can be precisely navigated to the position of the liver tissue by external fields. Upon the injection of cellulase via syringe, the HBSR microgripper can be actuated by the specific enzyme-substrate interactions and can clamp on tissue. With the assistance of magnetic fields, the liver tissues can be excised, and the microgripper can be retrieved. The methodology in this research work can be extended to other chemoresponsive micromotors. The prominent characteristics of their autonomous responses to disease markers or specific biochemicals endow the hydrogel-based chemoresponsive micromotors with great application potential in biological fields.

## 3. Conclusion and Future Outlook

This review summarizes the latest developments in stimuli-responsive medical micromotors, which are mainly composed of stimuli-sensitive hydrogels, actuation-responsive materials, and other functional components. Inheriting from the intrinsic properties of hydrogels, HBSR micromotors are biocompatible, biodegradable, flexible, and shape transformable in response to external environmental stimuli (such as pH, temperature, light, magnetocaloric, ion strength, and chemicals). The stimuli-induced swelling/deswelling behaviors of HBSR micromotors can be utilized for object manipulation at the microscale. In combination with external actuation systems and visual imaging techniques, the HBSR micromotors hold great promise in completing complex medical tasks (such as controlled loading and release of diagnostic and therapeutic reagents, tissue excision for biopsy, and removal of foreign bodies or abnormal cells) in physiological conditions. However, there is a long way to go before the hydrogel-based stimuli-responsive micromotors can be put into practical clinic applications.

The main directions for the future development of medical HBSR micromotors are as follows:

1. Programmable fabrication techniques of HBSR micromotors with more degrees of freedom for microsized object manipulation are highly needed. Currently, to generate nonuniform deformation, the architectures of HBSR micromotors are usually fabricated to possess heterogeneous features. Bilayer structures that combine two types of polymers (at least one polymer is stimuli-sensitive hydrogel) with different modulus [49, 50, 72] are widely used. However, the introduction of biologically incompatible or nondegradable components may cause undesirable physiological effects. Researchers have attempted to create the inhomogeneous structure of HBSR micromotors by precisely controlling the density distribution of a single hydrogel material in a whole architecture [62, 68]
2. The cycling times of swelling/deswelling determine the service life of HBSR micromotors, which is supposed to be a key performance parameter. Unfortunately, there are very few research reports on this performance metric
3. Although simple shape morphing of the HBSR micromotors can be achieved, it is still very challenging to carry out a collaborative shape deformation by employing multiple parts, mimicking the movement of living organisms in nature. For example, the swimming, hopping, and overturning of a fish in the water require the cooperation of all parts of its body (such as head, tail, and fin). The HBSR micromotors with more sophisticated shape-morphing capacities, which need more complicated microarchitectures, are expected to unlock more locomotion modes
4. If the stimuli arise from external fields (such as light, ultrasound, and magnetic field), the applied intensity, exposure time, and other parameters must be optimized to minimize the damage to organisms by these fields. Under ideal conditions, the stimuli should come from the localized changes in the physiological environment, such as the change of pH/temperature or the generation of new chemicals (e.g., proteases naturally secreted from tumor cells) in the diseased sites. Considering the exclusive and autonomous responsiveness to cancerous or other abnormal microenvironments by choosing suitable HBSR micromotors, it is envisioned that the HBSR micromotors can be exploited to carry out in situ diagnosis and therapy with a high spatiotemporal resolution. In case it is really necessary to introduce foreign stimulus signals (e.g., chemical stimuli, ionic strength stimuli, and pH stimuli) by injecting certain solutions into the body through a syringe or other tools, researchers must make sure that the exogenous solutions do not have any side effects on the healthy tissues and organs. The balance of the original physiological environment is only allowed to be broken in a tiny localized region (e.g., diseased sites)
5. Current researches only adopt in vitro or ex vivo experiments to confirm the feasibility of applying HBSR micromotors in targeted drug delivery, biopsy, removal of blood clots, and so forth. Here, we strongly encourage scientists to conduct more in vivo experiments to manipulate and navigate HBSR micromotors in the living environment, with the assistance of external fields (e.g., magnetic field, light, and ultrasonic field) and clinical imaging systems (e.g., magnetic resonance imaging and ultrasound imaging). The systematic preclinical studies on HBSR micromotors are the prerequisites for achieving their bench-to-bedside translation in the future

## Data Availability

Data of this paper are available by emailing lijinhua@bit.edu.cn.
